# Palaeognath birds innovate to solve a novel foraging problem

**DOI:** 10.1038/s41598-025-88217-8

**Published:** 2025-02-20

**Authors:** Fay E. Clark, Jasmine Burdass, Annalise Kavanagh, Annabel King

**Affiliations:** School of Psychological Science, University of Bristol, Bristol, BS8 1TU UK

**Keywords:** Animal cognition, Ratite, Technical intelligence, Psychology, Evolution, Zoology

## Abstract

The ability to innovate implies flexible cognition, and is used as a broad metric of intelligence. Innovation in birds has been intensively studied in the larger and more taxonomically diverse Neognathae clade (particularly crows and parrots) and overlooked in the smaller and more ancestral Palaeognathae clade. The current study provides the first known evidence of technical innovation in palaeognath birds. We tested the ability of nine individuals of three species to move a hole towards a chamber to access a food reward. This problem was different to traditional innovation puzzle-boxes where an obstacle is moved away from a food chamber. Three emus and one rhea produced a wheel-turning innovation, moving the hole in the most efficient direction (closer to the nearest food item) in 90% of cases. One rhea dismantled the task twice by removing the central bolt, which we suggest is a second type of innovation, and it did not persist once they innovated the wheel turning solution. Ostriches did not innovate. We classify innovation in palaeognaths as low level/simplistic, relying on general exploration and asocial trial and error learning. Our research suggests that technical innovation may have evolved far earlier in birds than previously thought, and palaeognath birds are a compelling taxonomic group for further cognitive research.

## Introduction

Innovation is defined as the use of pre-existing behaviours in novel circumstances or invention of novel behaviours^1,2^. Innovation has been a popular topic within animal cognition^1–3^, including its relationships to social learning^4,5^, behavioural plasticity^6,7^, and as a metric of general intelligence^8–10^. Inter- and intra-specific variation in innovation has received intensive study in birds. Large-scale analyses indicate that bird feeding innovations are robustly associated with larger relative brain and forebrain sizes, even when controlling for unequal numbers of species per taxon^4,11,12^. Such top-down analyses have been criticised for focussing on the diversity rather than context of innovations^13^. To address this, tasks have been used to assess how a standardised problem is solved by individuals, groups, species or taxa as a function of time (experience) along with predictors such as exploratory style, neophobia, age and sex^14^.

Palaeognaths (meaning ‘old jaws’) are the smaller and more ancestral of the two living bird clades and share more morphological features with extinct birds and extinct (non-avian) dinosaurs^15,16^. Palaeognaths include the flightless ratites (emu, *Dromaius novaehollandiae*; rhea, Genus *Rhea*; ostrich, Genus *Struthio*; cassowary, Genus *Casuarius*; and kiwi, Genus *Apteryx*), as well as the tinamous (family Tinamidae) which are poor at flight. Palaeognaths have the smallest relative brain size and relative forebrain size of all birds^16–18^ and this has been linked to a relative lack of evolutionary diversification^19,20^ and lack of parental investment^18^. Interestingly, ratites and tinamous have repeatedly lost their ability to fly^21^ and there is no clear relationship between flightlessness and brain size^22^ Although the ostrich has the largest absolute brain of all living birds, its brain is much smaller than predicted for body size (brain size 40.25 g, body size 120.05 kg)^17^. Furthermore, specific areas of the avian brain linked to cognition such as the pallium are reduced in size, complexity or neural activity in palaeognaths^17,23^. The only palaeognath species that defies this trend is the kiwi; it has a relatively large forebrain which may have evolved due to the strong sensory and perceptual pressures of nocturnality^24^.

Even though palaeognaths have been popularised as ‘bird-brained’ and lacking in intelligence, very little is known about their cognitive abilities. In a recent analysis of innovation, brain size and neuron number across 111 bird species, only one ‘feeding innovation’ (i.e., ability to access novel food)^17^ was recorded for each of three palaeognath species: emu *Dromaius novaehollandiae*, greater rhea *Rhea americana,* and common ostrich *Struthio camelus*^17^. There is no current evidence these species can use novel behaviour to access food (‘technical innovation’)^17^. In comparison, crows of the Genus *Corvus* had 59 innovations (*C. corax*: 35 total, 19 technical*; C. frugilegus*: 13 overall, 8 technical*, C. monedula*: 11 total, 4 technical)*.* It is currently unclear whether the lack of evidence for technical innovation in palaeognaths^17,25^ is due to a lack of cognitive ability or lack of research.

The current study will contribute important foundational knowledge on palaeognath physical cognition, which has until now been overlooked. Because they are the more ancestral living bird clade, Palaeognathae has been proposed as a cognitive model for non-avian dinosaurs and research has recently begun to establish their sociocognitive skills^26,27^. Greater rheas exhibit locomotor and social play^26^ but do not socially groom like other bird groups^28^. Intriguingly, ratites can follow the gaze of conspecifics^27^ a skill which is on par with some primates^29^.

In this study, we examined the ability of three palaeognath ratite species (emu, greater rhea, and common ostrich) to innovate a solution to a foraging problem (the novel foraging task paradigm for technical innovation)^30,31^. A novel ‘rotary task’ required birds to move a hole in a wheel towards a chamber to access a food reward. We used a novel task in the current study for several reasons. First, the established puzzle-box for bird innovation is a transparent plastic box with doors that can be pushed, pulled or slid open^30–32^. If this box design was scaled up to the relative body size of palaeognaths it would need to be extremely large; this was not practical nor safe given the lack of neophobia and object manipulation data on palaeognaths. Second, the rotary task is more ecologically relevant than a puzzle where each food item can be removed using a separate action (push, pull, slide) and relies on less human interference to refill. Third, we anticipated the rotary task could be easily modified for other bird species after the current study for comparative data.

It is reasonable to assume palaeognaths are capable of technical innovation because there seems to be no minimum brain size required for birds to innovate^17,18^. In line with several other scholars^33–37^, we subscribe to the viewpoint an animal’s possible innovation outcomes lie on a spectrum from simple to complex. We predicted all three palaeognath species would be capable of simplistic, ‘low level’ technical innovation (i.e., simple, repeated motor actions, basic exploration rather than causal inference). While rheas and emus have a larger relative brain size than ostriches (brain mass divided by body mass: rheas and emus approx. 6.7 × 10^−4^, ostriches approx. 3.3 × 10^−3^)^17^, palaeognath brains are more similar to each other than to other species^17,18^ so we did not make any predictions about the relative performance of each palaeognath species.

## Results

All emus approached and contacted an open version of the task (no wheel attached) in a 30-min familiarization session preceding the problem-solving sessions (Table 1). All approaches/contacts took place within the first 15 min. R1 and O1 approached the task but no rheas or ostriches made task contact in the familiarization session. Across all birds, there was a very weak negative relationship contact neophobia change and (i) task use rate and (ii) trial number in the problem-solving sessions (Bayesian correlations, Table 2), meaning that the more their neophobia reduced from familiarization to testing, the more they used the task.

**Table 1:** Summary of novelty responses during the (a) familiarization session and (b) first problem-solving session by individual birds.

Subject	Approach			Contact		
	Latency	Neophobia score	Neophobia change	Latency	Neophobia score	Neophobia change
E1	(a) 8.60 (b) 0.10	(a) 0.29 (b) 0.003	↓28.7%	(a) 11.60 (b) 0.70	(a) 0.39 (b) 0.02	↓37.0%
E2	(a) 11.8 (b) 0.40	(a) 0.39 (b) 0.01	↓38.0%	(a) 12.00 (b) 5.52	(a) 0.4 (b) 0.18	↓22.0%
E3	(a) 0.28 (b) 2.43	(a) 0.01 (b) 0.08	↑7.0%	(a) 0.52 (b) 2.43	(a) 0.02 (b) 0.08	↑6.0%
R1	(a) 9.80 (b) 0.30	(a) 0.33 (b) 0.01	↓32.0%	(a) 30.00 (b) 0.32	(a) 1.0 (b) 0.10	↓90.0
O1	(a) 4.00 (b) 0.13	(a) 0.13 (b) 1.00	↑87.0%	(a) 30.00 (b) 30.00	(a) 1.0 (b) 1.0	0.0%
O2	(a) 30.00 (b) 9.35	(a) 1.00 (b) 0.31	↓69.0%	(a) 30.00 (b) 30.00	(a) 1.0 (b) 1.0	0.0%
O3	(a) 30.00 (b) 12.23	(a) 1.00 (b) 0.41	↓69.0%	(a) 30.00 (b) 30.00	(a) 1.0 (b) 1.0	0.0%
O4	(a) 30.00 (b) 18.65	(a) 1.00 (b) 0.62	↓38.0%	(a) 30.00 (b) 30.00	(a) 1.0 (b) 1.0	0.0%

Latencies are the time (mins) to first approach and contact the task. Neophobia scores are latencies converted into proportions of total session length (30 min). Neophobia change is the percentage difference between neophobia score in (a) and (b). Birds were scored a maximum 30 min latency if they failed to perform a behaviour.

**Table 2:** Correlation matrix of neophobia, task use and task performance.

	Task use rate	Task contact rate	Hole move rate	Trial number
Neophobia score (approach)	r = − 0.504 BF_01_ = 1.538	r = − 0.472 BF_01_ = 1.761	r = − 0.429 BF_01_ = 2.075	r = − 0.451 BF_01_ = 1.911
Neophobia change (approach)	r = − 0.094 BF_01_ = 3.992	r = − 0.072 BF_01_ = 4.042	r = − 0.087 BF_01_ = 4.009	r = − 0.451 BF_01_ = 1.911
Neophobia score (contact)	r_s_ = − 0.488 BF_01_ = 1.651	r_s_ = − 0.486 BF_01_ = 1.660	r_s_ = − 0.299 BF_01_ = 2.032	r_s_ = − 0.197 BF_01_ = 3.599
Neophobia change (contact)	r_s_ = − 0.636 BF_01_ = 0.729	r_s_ = − 0.544 BF_01_ = 1.270	r_s_ = − 0.554 BF_01_ = 1.205	r_s_ = − 0.197 BF_01_ = 0.515

Performed using Bayesian Pearson correlations. N = 9 birds.

All three emus and one rhea (R1, male) produced a solution/s to the rotary task within the first problem-solving session (Table 3). Emus and R1 rotated the hole clockwise or anticlockwise by pecking or biting it so that the hole aligned with a food chamber (wheel innovation, Supplementary video). This happened 52 times (emus: 42, mean = 14.00 ± 9.50; rhea: 10). Even though ostriches made contact with the wheel they never moved it. One rhea (R2, female) did not approach or contact the task during the study so this subject is excluded from the remaining results.

**Table 3:** Summary of individual bird performance on the rotary task.

Subject	Total use D: duration (min) T: number of trials	Task contact rate	Motor diversity	Time to initial innovation S: number of sessions T: number of trials L: latency (min)	
				Wheel solution	Bolt solution
E1	D: 42.70 T: 56	259/240	1a, 1b, 2a, 2b, 2c	S: 1 T: 9 L: 29.8	N/A
E2	D: 11.98 T: 20	51/240	1a, 1b, 2a, 2b, 2c	S: 5 T: 9 L: 126.6	N/A
E3	D: 10.03 T: 14	55/240	1a, 1b, 2a, 2b, 2c	S:2 T: 3 L: 34.2	N/A
R1	D: 18.37 T: 33	109/210	1a, 1b, 2a, 2b, 2c, 3a, 3b	S: 3 T: 7 L: 64.5	S: 1 T: 1 L: 4.9
O1	D: 1.13 T: 2	8/240	1a, 1b	N/A	
O2	D: 1.02 T: 8	4/240	1a, 1b		
O3	D: 0.20 T: 5	2/240	2 1a, 1b		
O4	D: 1.87 T: 7	6/240	2 1a, 1b		

Task use refers to a bird’s close observation or contact with any task part, with or without hole movement. Task contact rate is the total frequency divided by total session minutes, to account for unequal numbers of sessions between species. Motor diversity is described in Methods Table 5.

R1 accessed food from the task via a second solution that bypassed the intended task design and we classify it as a second technical innovation. R1 bit the head of the central bolt and ‘ratcheted’ its head from side to side multiple times. This loosened the bolt from the nut and released the wheel from the base to uncover all five food chambers (Supplementary video). This took place twice (session 1 trial 1: 4.9 min latency; session 3 trial 7: latency 3.5 min from start of session). Session 1 and 3 were on two consecutive days, meaning the two bolt removals took place approximately 24 h apart. The second bolt removal took place approximately 1 min before the first wheel innovation (session 3 trial 7: latency 4.5 min from start of session). In both instances, the loose bolt and wheel were immediately retrieved from the enclosure by a researcher and the task was put back together, bearing in mind this did not stall or prevent further task use by the bird because there were two copies of the task in the enclosure.

**Fig. 1:**
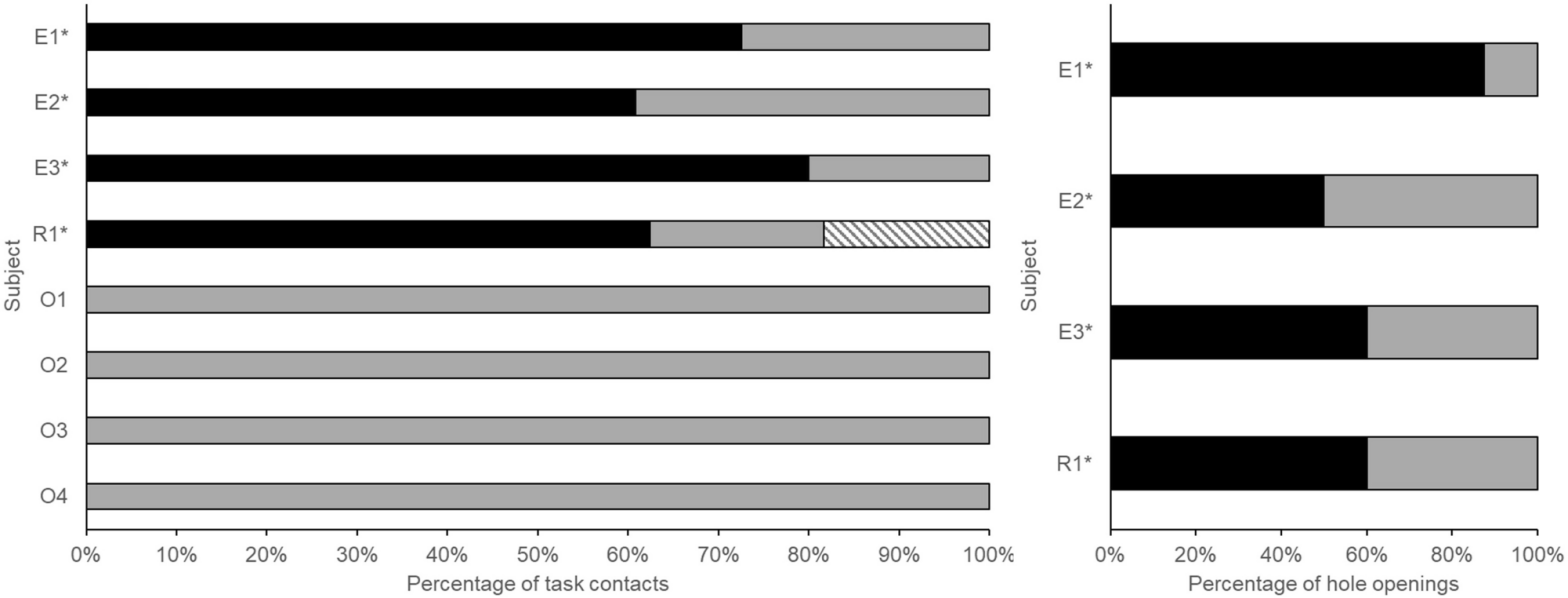
Proportions of biting, pecking and twisting by palaeognath birds using the rotary task. Left: task contacts (with and without hole movement). Right: hole openings. Black = bite, grey = peck, dashed = twist. Innovating individuals are shown by an asterix.

Of 391 total wheel contacts (all species pooled; including any contact to the wheel but not the cable ties or bolt etc.), 52/391 (13.3%) wheel contacts opened a food chamber. Emus (pooled data from N = 3 subjects) contacted the task 365 times over 8 sessions/90 trials (Table 3, Supplementary Table 1). 42 contacts (11.5%) resulted in the hole moving from an open to a closed position. Emus only moved the hole by biting (71.4% cases) or pecking (28.6% cases) the hole (Fig. 1). E1 (female) and E2 (male) both innovated in Trial 9. There was an effect of time (session number 1–4) on the number of task contacts (Bayesian repeated measures ANOVA, BF_01_ = 0.097, strong evidence for an effect of time) but this lost strength during pairwise Bayesian related samples t-tests (Day 1 vs 2: BF_01_ = 3.888, Day 1 vs 3: BF_01_ = 3.409, Day 1 vs 4: BF_01_ = 1.622, Day 2 vs 3: BF_01_ = 2.017, Day 2 vs 4: BF_01_ = 3.894, Day 3 vs 4: BF_01_ = 1.101). The same pattern of results was observed for hole moves (Bayesian repeated measures ANOVA, BF_01_ = 0.168, i.e., moderate evidence for an effect of time) which lost strength during pairwise Bayesian related samples t-tests (Day 1 vs 2: BF_01_ = 2.893, Day 1 vs 3: BF_01_ = 2.974, Day 1 vs 4: BF_01_ = 2.125, Day 2 vs 3: BF_01_ = -2.273, Day 2 vs 4: BF_01_ = 2.407, Day 3 vs 4: BF_01_ = 1.769).

**Fig. 2:**
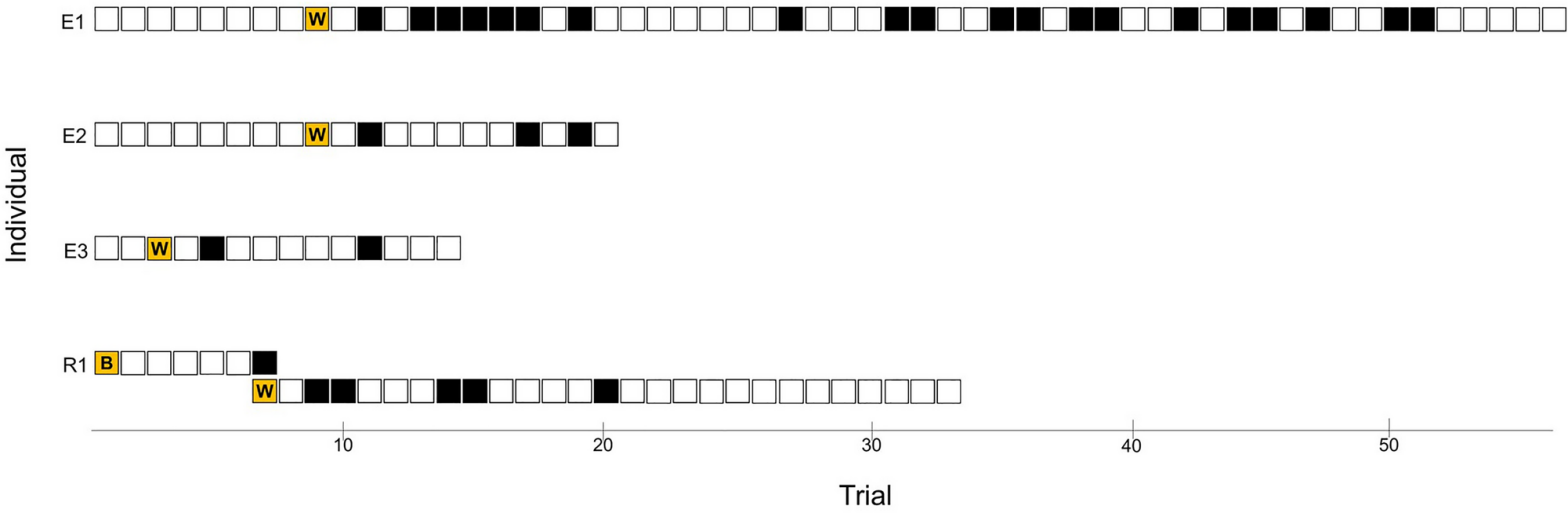
Time distribution of the first occurrence of innovative behaviour (yellow fill, W = wheel solution, B = bolt solution) and subsequent use of these behaviours (black fill) on the rotary task. Each square represents one trial, sessions are not shown for simplicity. In trial 7, bird R1 innovated the wheel solution and stopped using the bolt solution. Trials are shown rather than sessions for simplicity.

R1 contacted the task 109 times across 7 sessions (33 trials) and 10 (9.2%) of these resulted in the wheel moving from an open to a closed position (wheel solution). This was achieved by biting (40%) or pecking (60%) the hole. R1 also twisted the bolt to remove the wheel entirely (bolt innovation) on 2 occasions (Fig. 2, trial 1 and 7). Birds who innovated had a significantly higher motor diversity than birds who did not (Bayesian independent samples t-test, BF_01_ = 0.017, very strong evidence for an effect of motor diversity).

After performing the wheel innovation for the first time, all emus continued to use this behaviour in subsequent trials (mean 29.3 ± 7.1% subsequent trials contained at least one wheel innovation, Fig. 2). After innovating the bolt solution in the first session (trials 1 and 7), the R1 innovated the wheel solution (trial 7) and did not use the bolt solution again, thereby replacing one solution with another. The wheel solution was used in 19.2% of R1’s subsequent trials. In trials where the innovated behaviour was used (including the first case of the innovation), it was used on average 1–3 times per trial: E1 mean = 1.57 ± 0.27; E2 mean = 1.00 ± 0.00; E3 mean = 1.00 ± 0.67; R1 mean = 3.00 ± 0.33.

Emus and rheas moved the hole in 90.4% cases toward a chamber containing food, and in 9.6% cases toward an empty chamber. When a full chamber was opened, food was always extracted and consumed but sometimes birds let food drop to the floor before consuming it, and in rare cases only partially extracted food (so the number of extractions exceeded the number of items available, Table 4). There were individual and species differences in how many ‘free’ food extractions were made, in other words, how much food birds took from the open chambers at the beginning of each session during sessions when the task was re-filled (Table 4). Emus took all free food available to them (47/47 items across the problem-solving sessions), R1 took 46.7% (14/30) and the ostriches took 43.8% (7/16), indicating a lower motivation for lettuce in rheas and ostriches compared to emus. It is not possible to perform further analyses because the numbers of freely open chambers differed between species due to unequal session numbers and not being able to refill the ostrich task.

**Table 4:** Total food extractions by individual birds from the rotary task.

Subject	Familiarisation session	Problem-solving sessions		
	Total	Total	Free food	Occluded food
E1	24	81	48*	33
E2	1	8	4	4
E3	16	9	4	5
R1	0	24	14	10
O1	0	5	5	0
O2	0	0	0	0
O3	0	0	0	0
O4	0	2	2	0

Free food refers to food from already open food chambers, and occluded food refers to food occluded by the wheel. All food chambers were freely open in the familiarization session. In the problem-solving sessions, closed chambers could only opened by moving the hole.

*The total number of extractions from free chambers exceeded the number of free items available if a bird made several revisits to extract the same item. The ostrich task could not be refilled for safety reasons.

When a food chamber was opened, this was usually by 36° rotation of the hole from a closed to an open position to align with the nearest chamber (48/52 cases, 92.3%). Rarely, a bird moved the hole more than 72° from one open chamber to the next which meant they had to effectively close the hole before it opened again (4/52 cases, 7.7%). There was a lack of evidence of lateralized problem-solving in birds (pooled data for all three species). Birds made contact with the top five positions of the wheel more than the bottom five positions of the wheel (top: 232, bottom: 159 contacts) but there was only anecdotal evidence for an effect of height (Bayesian two-tailed binomial test, BF_01_ = 1.541). Clockwise and anticlockwise moves were roughly equal (clockwise: 22, anticlockwise: 30 moves, Bayesian two-tailed binomial test, BF_01_ = 1.538) and the number of contacts made to the left and right of the task (253 versus 221 contacts) was also similar (Bayesian two-tailed binomial test, BF_01_ = 1.566).

## Discussion

Large-brained crows and parrots have dominated the field of avian cognition^38^, but recently there has been a move towards studying rarer and smaller-brained species^39,40^. Our study provides empirical evidence of technical innovation in palaeognath birds, in the context of novel food extraction. Previously there has only been evidence of food innovation in palaeognaths, and large-scale top-down analyses have reinforced the message these birds have very small brains which has probably precluded cognitive research^16–18^. Our results are therefore a notable contribution to the literature. We found that four individual birds (three female emus and one male rhea) each produced one or more solutions to remove food from a physical task.

Even though in essence, all innovation tasks for birds and other species involve the extraction of food by somehow overcoming a physical barrier^14,30–32^, the rotary task was specifically designed to be solved by aligning a hole with food, rather than the entrenched paradigm in animal cognition which is to remove an obstacle covering the food. We suggest they are notably different; aligning a hole with food is ‘additive’ problem-solving (the hole and the food must be brought together) whereas removing an obstacle from food is ‘subtractive’ (the obstacle and food must be separated). Given the early stages of our research and because we did not test a traditional puzzle-box on palaeognaths for clear comparison with other species (which we appreciate would be the preferred approach of many scholars, particularly when working with new taxa), we suggest there is scope for further research on substantially additive versus subtractive problems within and across bird species. In particular, it would be interesting to develop a task where factors affecting additive *versus* subtractive problem-solving can be investigated.

The topic of animal innovation is vast, and starting with a blank slate in terms of palaeognath physical cognition we decided to focus on their innovation ability in terms of problem-solving on a task^1,30,31^. We therefore frame our results within two broad forms of innovation: the use of pre-existing behaviours in novel circumstances and the invention of novel behaviours^1,2^. All four innovating birds in our study (three emus and one rhea) used pre-existing behaviour in a novel circumstance: pecking/biting was already present in their behavioural repertoire to explore, feed, groom and so on, and was used to align a hole in a barrier with a food chamber (wheel innovation).

Regarding the bolt solution by the male rhea, there are several reasons to classify this as a second type of innovation. First, even though we did not intend for the task bolt to be removed (a small, loose item poses potential safety issues) animal innovation frequently occurs through ‘lucky accidents’^41^ where rudimentary behaviour leads to a reward without apparent intention to do so. Even if the bird acted upon the bolt without intending to access food, for example its shininess was visually appealing due to neophilia, this still classifies as low level innovation via general discovery through exploration^33–37^. Second, when accidental discoveries are actively used in future to reach the same outcome, this is further evidence of innovation because an association has been made between new behaviour and a beneficial outcome. The bolt removal occurred twice, in separate testing sessions. Third, the bolt removal behaviour was extinguished (removed from the repertoire) when the rhea innovated the wheel moving solution. We speculate this could be because wheel moving was physically easier than bolt removal. We could not find any report of palaeognaths head ratcheting in the wild or captivity, but cannot rule out the possibility R1 previously learned this behaviour in a different context and re-applied it here (e.g., perhaps it learned to successfully remove a bolt from a fence or feeder in its previous zoo).

We have presented evidence of technical innovation in two species of palaeognath bird, but remain cautious about how ‘impressive’ these innovations are, if we place them into the wider context of animal innovation. It is not yet possible to compare the performance of birds in our study to other species because the rotary task has only been used on palaeognaths. We predict crows and parrots will perform well on the task, given that food wheels are sold as pet enrichment and parrots can learn to remove food from rotating feeders within a few days with no training^42^. Rotating elements have been used in previous cognitive tasks but lack relevance to our task because animals have learned to align components of a pipe puzzle by rotating them on a turn-table (chimpanzees *Pan troglodytes*)^43^; or push a very large wheel multiple times greater than their own body length (bumblebees *Bombus terrestris*)^44^.

Following several previous conceptualisations of innovation on a spectrum from low level (simple) to high level (complex)^33–37^, we would describe the innovations observed in the current study as relatively low level and cognitively simple. While emus and the rhea repeated their innovated behaviours across eight testing sessions which rules out accidental occurrence and supports the concept of innovations having beneficial outcomes^1^, their innovations arose from basic exploration rather than causal inference^34^. We acknowledge that wheel movement did not require much physical effort, the task was available for prolonged periods (not traditional time-restricted trials) and there were no motivational pressures like food deprivation^35–37^. If we set a theoretical bar at crow and parrot standards it would be tempting to dismiss palaeognath task-use as ‘unimpressive’. Albeit simple, palaeognaths in this study do meet the definition of technical innovation^1,2^ which is a highly novel finding in bird cognition, mirroring recently discovered innovation skills in other small-brained taxa (such as bat-eared foxes *Otocyon megalotis*, the smallest-brained canid)^45^.

Animal cognition researchers often debate whether an animal’s behaviour is due to learning, cognition or both^46^. Our primary aim was to see if palaeognaths could innovate rather than precisely how, namely because our resources made it impractical to perform highly controlled experiments on these birds. However, we can propose how the palaeognath innovation arose. It seems likely that the wheel-moving innovation occurred through individual trial and error learning, which corresponds to our overall interpretation of low level/simple innovation. Birds independently learned the association between a motor action (e.g., pecking, biting) and a desirable outcome (i.e., hole movement) and potentially some additional perceptual motor feedback from the wheel moving^47,48^. Unlike many classic task apparatuses where one action is scored as a pass or fail, birds could perform many beak-to-task contacts in a row on the rotary task, which allowed rapid operant learning. Furthermore, this is notably different from traditional puzzle-boxes where there is one major action (e.g., push a lid, pull a lid). Repeatedly pecking or biting at the hole gradually revealed food, similar to how a bird in a string-pulling task can incrementally move the food closer to its feet by repeated grasping^47^. Hole moving was deliberate rather than random because birds aimed their responses at open food and the hole, rather than food behind the barrier. They also moved the hole in the most efficient direction (closer to the nearest food item) in 90.4% of cases.

Even though we found a positive association between motor diversity and innovation ability in palaeognaths which is supported by previous avian research^49^, a wide range of motor actions were not required to solve the rotary task. It is therefore intriguing why the male rhea’s first innovation was more complex than the second, and again raises the question of whether bolt-ratcheting was already in its repertoire (at the zoo it lived at three months prior to our study). The animals in this study were not kept under constant observation under controlled conditions, so we cannot know the provenance of such behaviours. It is surprising the bolt innovation occurred so rapidly, within the first 5 min of the rhea’s first test session, and without any physical task exploration in the familiarisation session. This bird’s lack of prior task experience rules out innovation through insight (i.e., a sudden and pre-informed solution, presumed to arise from cognitive re-structuring of the problem following an impasse)^50^ so points towards using a previously learned behaviour (albeit a complex one) in a novel situation. We also assume there was an element of playful interaction, similar to striated caracaras (*Phalcoboenus australis*) whose interaction with a puzzle-box was reinforced by task sound or movement^40^. However, if the rapid bolt innovation had been witnessed in a crow or parrot, we suspect it would have been acceptable, perhaps expected of us, to attribute it to insight or causal reasoning, based on prior findings in these taxa^38^. This serves as a reminder to animal cognition researchers to consider simplistic explanations for behaviour first, even in more advanced species^51^.

Some scholars will question why we baited the task with ‘free’ (unoccluded) food throughout the study because it might confuse or dilute birds learning the association between hole movement and food access. Almost nothing is known about the perceptual abilities of palaeognaths, including if they can perceive food behind a transparent barrier, so we felt it necessary to facilitate associative learning of task presence and food presence. Of more concern, and only with hindsight, was some of the rotary task design features. A wheel that moved in either direction made it possible for a bird to move the hole away from the closest food item but still eventually encounter another food item by chance. As stated previously, emus and the rhea nearly always moved the wheel in the most efficient direction, but a non-circular task design (i.e., with dead ends) would make the deliberateness of their actions easier to score. We also recommend a design with a clearer distinction between ‘pulling’ a hole towards a food chamber, or ‘pushing’ a solid barrier away from a food chamber, and therefore our aforementioned suggestion for comparisons between additive and subtractive problem-solving.

There were clear inter-species and inter-individual differences in problem-solving and innovation in this study, which is expected yet difficult to identify causal factors for given our small sample size^52^. Small-scale studies like ours have widely been used in comparative cognition as initial probes of concepts rather than rigorous tests^53^, and we did not aim to identify robust predictors of problem-solving and innovation. However, it is notable that ostriches were incapable of innovation within the parameters of the rotary task. This was likely due to low motivation and neophobia, and potential issues with the task dimensions. There was a substantial size difference between our smallest species (rhea, 1.5–1.7 m tall) and largest species (ostrich, 2.5-3 m tall)^54^ that may have placed more postural or beak limitations on ostriches. Further research is required to establish whether ostriches show consistently lower performance; a logical hypothesis (but highly speculative at this stage) is that ostriches have the poorest performance because they have the smallest relative brain size and are the most taxonomically distant^55,56^.

Similar to previous research on birds^30,57^ we found that individuals had their own problem-solving ‘paths to success’ and apart from motor diversity, there were no clear relationships between any metrics of neophobia, task-use or task success. Although innovating birds continued to use innovated behaviour throughout the study, success and engagement fluctuated from session to session. Another interesting observation is that the female emu E3 who performed an oral stereotypy (air-biting) prior to the study performed this behaviour within 5 cm of the task. We do not have data to investigate whether the rotary task was perceived as a stressor by birds, but there was no lateralised behaviour such as a task side bias or preference for moving the hole in one direction. This is interesting because side and body biases have previously been identified in primates under negative stress in cognitive experiments^58,59^.

Finally, we briefly consider wider research implications, being mindful that our study was an initial step into some unknown territory. The majority of research on palaeognath behaviour to date has been restricted to their use as farmed animals; therefore, our study is an important move towards targeted cognitive research. Not only does it contribute knowledge on previously overlooked bird species, it also supports theoretically using paleognaths as living models for non-avian (extinct) dinosaur behaviour and cognition, in conjunction with recent work on their sociocognitive skills^26–28^. We note that because dinosaur skulls (more specifically their endocasts) are poor proxies for the size and structure of the living brain^60^, there has been growing interest in observing the skills of live birds with similar characteristics to dinosaurs^26–28^. However, there is still much work to be done to assess the true utility of these proxies^61^. We expect some palaeognaths will emerge as better proxies than others, not merely due to phylogeny but also due to their size, ease of management and motivation to engage with cognitive tasks.

## Methods

### Subjects

Subjects were three emus, two rheas and four ostriches housed at Noah’s Ark Zoo Farm, United Kingdom. All subjects were adults. The age of the female rhea (R2) was estimated by keepers because it was rescued from private land four years ago, presumed to be a pet (Table 5). Emus and rheas were housed in single-species enclosures and ostriches shared part of their enclosure with Grant’s zebras (*Equus quagga boehmi*) and giraffes (*Giraffa camelopardalis*) but the ungulates could not access the tasks. All species were in outdoor paddocks and had free access to water, grass, trees and shrubs for grazing. They were provided with fresh commercial pellet 30–60 min following the end of trials. At the time of testing, no subjects in our study had participated in any other cognitive experiment to our knowledge. Task-use was non-invasive and subject’s participation was entirely voluntary. Tasks did not block or otherwise interfere with key feeding or resting areas, birds were tested socially in their normal enclosures and not during any known breeding period. Task food was taken from each species’ daily ration and returned to the ration after a session if unused.

**Table 5:** Subject information.

Subject	ID	Sex	Age (years)	Duration of housing at zoo (years)	Genetic relationship
Emu	E1	F	14	14	Siblings
	E2	M	14	14	
	E3	F	14	14	
Rhea	R1	M	3	0.25	Unrelated
	R2	F	6 (estimated)	4	
Ostrich	O1	F	11	9	Mother (O1) and offspring
	O2	F	5	5	
	O3	F	5	5	
	O4	F	5	5	

### Task

**Fig. 3:**
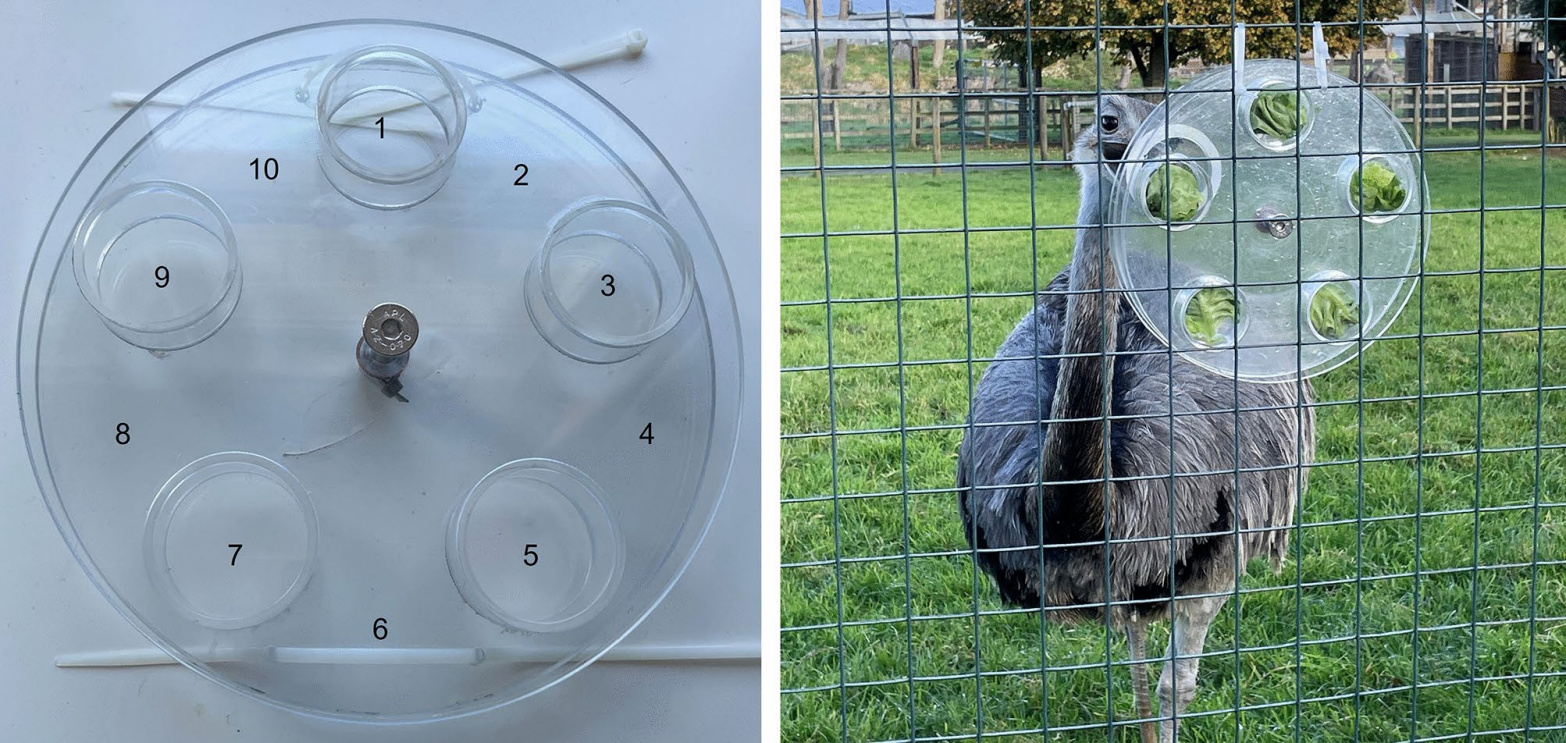
The rotary task. Left: a number scoring system to measure the direction and degree of hole movement. Odd numbers: food chambers, even numbers: spaces between chambers, numbered clockwise from the bird’s point of view. Right: the task in situ with rhea R1.

The task (Fig. 3) was constructed from transparent acrylic plastic (0.5 cm thickness). It had a circular base with five equally spaced circular food chambers (base: 26 cm ⌀, chambers: 6 cm ⌀, 2.5 cm deep), and a wheel with one circular hole towards the edge (wheel: 6 cm ⌀, hole: 60 mm ⌀). The rim of the hole was painted white (1 cm border) so that it was visible against the transparent background. The wheel and base were held together by a steel nut and bolt (0.8 cm ⌀, 8.5 cm length) running through the centre. The bolt was sunken into the base so that the base remained static when the wheel rotated. The tension on the bolt was adjusted by hand with a wrench so that bolt felt secure; it was loose enough to prevent cracking the wheel but tight enough so that the wheel did not spin freely without being touched.

Food could be accessed from the rotary task by aligning the hole over the top of a food chamber. It was not possible for birds to remove food from a chamber until they could insert their open beak, so a chamber that was at least 75% aligned with the hole was scored as open. The task was appropriate for the birds’ morphology because palaeognaths use directional pecking, plucking and biting during normal foraging, and often forage from vertical substrates like trees and shrubs^62^. The task required innovation because subjects were naïve to the task and, more broadly, naïve to the concept of food located behind a solid rotating barrier.

The rotary task was different to prior innovation puzzle box designs (that involve moving an obstacle away from a food chamber; for example by manipulating a door)^14,32^ and we hoped this would provide novel insights into innovation. The modular design was beneficial because it offered birds several food extraction opportunities in a row before it needed to be refilled, which is more representative of foraging in a natural food patch^40^. To this end, we defined a trial as a continuous period of voluntary task-use beginning with a bird’s approach within one body length and ending as soon as the bird retreated over one body length and did not return to use the task within 1 min.

## Experiment

Testing took place between October and November 2023. Each bird species was tested on five weekdays (emus: five non-consecutive days over a 10 day period to avoid a public holiday; rheas and ostriches: 5 consecutive days). Emus were tested first, then rheas, then ostriches for logistical reasons. One rhea session (session 8) was called off due to stormy weather; therefore there was one less session for this species and data are presented as relative values where appropriate. Two identical copies of the task were placed vertically on the fence using cable ties. This aimed to maximize the amount of potential data collection per session but birds never monopolized or displaced each other from the tasks.

Food chambers were filled with one leaf of butterhead lettuce because it was a preferred dietary item for all species and did not crumble into pieces when handled. Due to living in a zoo, all species were habituated to human presence and noise at the fence lines. Therefore it was possible for up to three experimenters to stand behind the emu and rhea fences during a session, maintaining low conversation throughout. Due to zoo rules, it was not possible to stand behind the ostrich fence or refill the task.

For each species, day 1 was a 30 min familiarization session where the birds were exposed to the task without the wheel and could freely remove food from the open chambers. This was an initial test of neophobia and an opportunity for birds to gather pertinent information prior to problem-solving^39,45^. For emus, task food was re-filled by an experimenter as needed throughout the session, at opportunities when the birds were at least one body length away and their heads were turned away. The rhea and ostrich tasks were not refilled because rheas did not extract food during the familiarization trial, and the ostrich task could not be refilled due to safety rules.

After the familiarization session, eight problem-solving sessions were run per species, two sessions per day, one AM (starting approx. 10:30 h) and one PM (starting approx. 12:30 h). In the problem-solving sessions, the task wheel was attached to the base with a nut and bolt (Fig. 3). Two copies of the task were presented for 30 min. The session began with each task having one open chamber (randomly assigned) and opportunity to extract one food item for ‘free’, similar to the familiarization session, without needing to move the hole. During a session, an experimenter refilled emptied chambers opportunistically, when birds were faced away from the task. After refilling, the location of the hole was moved by an experimenter to a random closed or open position, therefore sometimes offering more opportunities to extract a free food item. A session began with all food chambers filled, and ended automatically after 30 min, even if birds did not use the task for a period of time.

## Video coding and analysis

All sessions were videotaped and coded by one of four experimenters, with 10% double-coded to assure inter-observer reliability (over 90% agreement). Files were randomly allocated to each experimenter and coded in random order to minimize coding bias. A bird’s behaviour was coded whenever they were within one body length of the task, measured approximately from tail to beak in an upright standing position.

In the familiarization session, we scored the time it took each bird to approach within one body length (approach latency), and contact the task (contact latency). To account for different enclosure sizes and the fact emus tended to be closer to the fence than rheas and ostriches when a session started, latency was timed from when a bird was within three body lengths of the task. If a bird did not approach or contact the task within 30 min it was assigned the maximum value of 30 min because this was a response failure rather than missing data^39^. To assess individual birds’ responses to novelty (the first exposure to the open task in the familiarization session), we calculated neophobia scores by converting latencies into proportions of the time available; for example, a bird approaching the task at 5 min would be scored 5/30 min = 0.17. Larger values approaching 1.0 represent greater neophobia. We also calculated the percentage change in neophobia score between the familiarization session and first problem-solving session. Larger percentage values represent a greater change in neophobic response between the familiarization session and first problem-solving session.

Task use was quantified as a total duration and total frequency of contacts per session per bird (also referred to as persistence or ‘work time’ in the literature)^14,39^. In the problem-solving sessions, we recorded all behaviours used to contact the task, in addition to looking (Table 6). A motor diversity score was calculated for each bird as the total number of different motor behaviours used on the task. Because these species make singular, discrete head movements it was appropriate to score one peck/bite as one beak-to-task contact^48^. We did not count food extraction or feeding following food extraction within the motor diversity score. We also did not include stereotypical behaviour (rhythmically biting the air, E3 only) as a form of motor diversity because while this behaviour was sometimes directed towards the task it did not make contact. We recorded any outcome/s of behaviour (hole movement and/or food extraction).

**Table 6:** Description of motor actions during innovative problem-solving.

Behaviour	Task component		
	Hole	Bolt	Other component
1. Peck	a. Contact the task hole with a closed beak	b. Contact the bolt with a closed beak	See a-b, at the wheel surface, side of wheel or cable ties
2. Bite	a. Contact the edge of the task hole with an open beak	b. Contact the bolt with an open beak	
3. Twist	N/A	a. Contact the bolt with an open beak and repeatedly twist (ratchet) the head left to right	

Every time the wheel was contacted, any hole movement was quantified by recording its start and end location as one of 10 discrete positions, similar to compass points, which also captured whether the movement was clockwise or anticlockwise (from the bird’s point of view; Fig. 2). Food chambers were labelled 1,3,5,7,9 clockwise from the top, and the spaces between them were labelled 2,4,6,8,10. When the hole moved from open to closed or vice versa, for example from location 1 to 2, this was equivalent to 36 degrees of rotation (360 degrees/10 positions).

All statistics were undertaken in SPSS version 29.0.2.0(20) and means are presented ± standard error. To examine changes in problem-solving behaviour as a function of experience (time), we merged data per test day (the AM and PM session) and conducted a Bayesian repeated measures ANOVA and post hoc Bayesian related samples t-tests over four test days with numbers of task contacts and food chambers opened as separate dependent variables, with uninformative priors. Neophobia scores, proportional neophobia change (approaches and contacts) and motor diversity score were correlated against measures of task use and task performance using Bayesian correlations with a default prior. Bayesian two-tailed binomial tests with priors set at 0.5 were used to analyse differences in frequencies of wheel turns and task locations contacted. A Bayes factors (BF_01_) greater than 3 was taken as substantial evidence to support the null hypothesis (i.e., no effect), and a Bayes factor less than 0.33 was taken as substantial evidence for the alternative hypothesis (following Bayesian analysis performed by Bastos et al.^63^.

## Supplementary Information

Supplementary Information 1.

Supplementary Video 1.

## Data Availability

Supporting data is available as electronic supplementary material. Further raw data is available upon request from the principal author.
