# Supplementary material for: Palaeognath birds innovate to solve a novel foraging problem
Source: Sci Rep. 2025 Feb 20;15:4512. doi: 10.1038/s41598-025-88217-8 (PMC11842627; doi:10.1038/s41598-025-88217-8)
Supplement: Supplementary file 1 — Supplementary Information 1. [file 41598_2025_88217_MOESM1_ESM.docx]

Supplementary Table 1. Summary table of rotary task use by individual birds. (a) Number of trials, (b) task-use duration (min), (c) number of task contacts, and (d) numbers of food chamber openings per bird. Innovating individuals are shown by an asterix.

| **Subject** | **Session** | | | | | | | |  |
| --- | --- | --- | --- | --- | --- | --- | --- | --- | --- |
|  | **1** | **2** | **3** | **4** | **5** | **6** | **7** | **8** | **Sum** |
| E1* | (a) 9  (b) 0.90  (c) 33  (d) 2 | (a) 8  (b) 4.05  (c) 59  (d) 10 | (a) 7  (b) 3.97  (c) 10  (d) 1 | (a) 3  (b) 3.78  (c) 16  (d) 6 | (a) 8  (b) 12.00  (c) 30  (d) 4 | (a) 7  (b) 10.12  (c) 42  (d) 4 | (a) 7  (b) 5.15  (c) 32  (d) 4 | (a) 7  (b) 2.73  (c) 37  (d) 2 | (a) 56  (b) 42.70  (c) 259  (d) 33 |
| E2* | (a) 3  (b) 0.25  (c) 6  (d) 0 | (a) 4  (b) 0.53  (c) 8  (d) 0 | (a) 1  (b) 0.28  (c) 2  (d) 0 | (a) 0  (b) 0.00  (c) 0  (d) 0 | (a) 5  (b) 4.05  (c) 11  (d) 2 | (a) 3  (b) 0.42  (c) 6  (d) 0 | (a) 2  (b) 2.02  (c) 9  (d) 1 | (a) 2  (b) 4.33  (c) 9  (d) 1 | (a) 20  (b) 11.88  (c) 51  (d) 4 |
| E3* | (a) 2  (b) 0.10  (c) 4  (d) 0 | (a) 1  (b) 1.30  (c) 12  (d) 3 | (a) 1  (b) 0.23  (c) 1  (d) 0 | (a) 1  (b) 2.05  (c) 10  (d) 1 | (a) 4  (b) 1.30  (c) 7  (d) 0 | (a) 3  (b) 2.85  (c) 12  (d) 0 | (a) 1  (b) 1.48  (c) 8  (d) 1 | (a) 1  (b) 0.72  (c) 1  (d) 0 | (a) 14  (b) 10.03  (c) 55  (d) 5 |
| R1* | (a) 3  (b) 1.58  (c) 15  (d) 1^B^ | (a) 3  (b) 0.62  (c) 2  (d) 0 | (a) 5  (b) 10.33  (c) 45  (d) 1^B^, 5^W^ | (a) 1  (b) 0.13  (c) 2  (d) 0 | (a) 6  (b) 1.60  (c) 13  (d) 3^W^ | (a) 7  (b) 3.28  (c) 25  (d) 2^W^ | (a) 8  (b) 0.82  (c) 7  (d) 0 | N/A | (a) 33  (b) 18.37  (c) 109  (d) 2^B^, 10^W^ |
| O1 | (a) 0  (b) 0.00  (c) 0  (d) 0 | (a) 0  (b) 0.00  (c) 0  (d) 0 | (a) 0  (b) 0.00  (c) 0  (d) 0 | (a) 1  (b) 0.32  (c) 4  (d) 0 | (a) 0  (b) 0.00  (c) 0  (d) 0 | (a) 1  (b) 0.82  (c) 4  (d) 0 | (a) 0  (b) 0.00  (c) 0  (d) 0 | (a) 0  (b) 0.00  (c) 0  (d) 0 | (a) 2  (b) 1.13  (c) 8  (d) 0 |
| O2 | (a) 2  (b) 0.43  (c) 0  (d) 0 | (a) 1  (b) 0.07  (c) 0  (d) 0 | (a) 1  (b) 0.07  (c) 0  (d) 0 | (a) 2  (b) 0.25  (c) 2  (d) 0 | (a) 0  (b) 0.00  (c) 0  (d) 0 | (a) 2  (b) 0.20  (c) 2  (d) 0 | (a) 0  (b) 0.00  (c) 0  (d) 0 | (a) 0  (b) 0.00  (c) 0  (d) 0 | (a) 8  (b) 1.02  (c) 4  (d) 0 |
| O3 | (a) 2  (b) 0.08  (c) 0  (d) 0 | (a) 0  (b) 0.00  (c) 0  (d) 0 | (a) 0  (b) 0.00  (c) 0  (d) 0 | (a) 2  (b) 0.08  (c) 1  (d) 0 | (a) 0  (b) 0.00  (c) 0  (d) 0 | (a) 1  (b) 0.03  (c) 1  (d) 0 | (a) 0  (b) 0.00  (c) 0  (d) 0 | (a) 0  (b) 0.00  (c) 0  (d) 0 | (a) 5  (b) 0.20  (c) 2  (d) 0 |
| O4 | (a) 1  (b) 0.08  (c) 0  (d) 0 | (a) 2  (b) 1.40  (c) 0  (d) 0 | (a) 0  (b) 0.00  (c) 0  (d) 0 | (a) 2  (b) 0.13  (c) 3  (d) 0 | (a) 0  (b) 0.00  (c) 0  (d) 0 | (a) 2  (b) 0.25  (c) 3  (d) 0 | (a) 0  (b) 0.00  (c) 0  (d) 0 | (a) 0  (b) 0.00  (c) 0  (d) 0 | (a) 7  (b) 1.87  (c) 6  (d) 0 |
